# Insufficient Radiofrequency Ablation Promotes Angiogenesis of Residual Hepatocellular Carcinoma via HIF-1α/VEGFA

**DOI:** 10.1371/journal.pone.0037266

**Published:** 2012-05-15

**Authors:** Jian Kong, Jinge Kong, Bing Pan, Shan Ke, Shuying Dong, Xiuli Li, Aimin Zhou, Lemin Zheng, Wen-bing Sun

**Affiliations:** 1 Department of Hepatobiliary Surgery, West Campus, Capital Medical University, Beijing Chao-yang Hospital, Beijing, People's Republic of China; 2 The Institute of Cardiovascular Sciences and Institute of Systems Biomedicine, School of Basic Medical Sciences, and Key Laboratory of Molecular Cardiovascular Sciences, Peking University Health Science Center, Ministry of Education, Beijing, People's Republic of China; 3 Department of Gynecology and Obstetrics, the Chinese PLA General Hospital, Beijing, People's Republic of China; 4 Clinical Chemistry Program, Department Of Chemistry, Cleveland State University, Cleveland, Ohio, United States of America; University of Colorado, United States of America

## Abstract

**Background:**

The mechanism of rapid growth of the residual tumor after radiofrequency (RF) ablation is poorly understood. In this study, we investigated the effect of hyperthermia on HepG2 cells and generated a subline with enhanced viability and dys-regulated angiogenesis in vivo, which was used as a model to further determine the molecular mechanism of the rapid growth of residual HCC after RF ablation.

**Methodology/Principal Findings:**

Heat treatment was used to establish sublines of HepG2 cells. A subline (HepG2 k) with a relatively higher viability and significant heat tolerance was selected. The cellular protein levels of VEGFA, HIF-1α and p-Akt, VEGFA mRNA and secreted VEGFA were measured, and all of these were up-regulated in this subline compared to parental HepG2 cells. HIF-1α inhibitor YC-1 and VEGFA siRNA inhibited the high viability of the subline. The conditioned media from the subline exerted stronger pro-angiogenic effects. Bevacizumab, VEGFA siRNA and YC-1 inhibited proangiogenic effects of the conditioned media of HepG2 k cells and abolished the difference between parental HepG2 cells and HepG2 k cells. For *in vivo* studies, a nude mouse model was used, and the efficacy of bavacizumab was determined. HepG2 k tumor had stronger pro-angiogenic effects than parental HepG2 tumor. Bevacizumab could inhibit the tumor growth and angiogenesis, and also eliminate the difference in tumor growth and angiogenesis between parental HepG2 tumor and HepG2 k tumor *in vivo*.

**Conclusions/Significance:**

The angiogenesis induced by HIF1α/VEGFA produced by altered cells after hyperthermia treatment may play an important role in the rapid growth of residual HCC after RF ablation. Bevacizumab may be a good candidate drug for preventing and treating the process.

## Introduction

Hepatocellular carcinoma (HCC) is the fifth most common cancer worldwide and the third most common cause of cancer mortality [1]. Although hepatic resection and transplantation have been considered as the main curative therapies for HCC, the advanced neoplastic stage, severity of liver diseases or shortage of donors limit their application [2]. Recently, Loco-regional treatments play a key role in the management of HCC [3], especially radiofrequency (RF) ablation because of its definitive therapeutic effect, minimal invasiveness, repeatability and safety [2]. RF ablation heats tissues by ionic friction from RF current, which induces coagulation necrosis once tissue temperature exceeds 50°C for 4–6 minutes [3].

However, more and more clinical cases about rapid growth of residual HCC after RF ablation have been reported since 2001 [4]–[7]. RF ablation of colorectal liver metastases promotes proliferation of residual intra-hepatic neoplastic cells [8]–[11]. Our previous research also demonstrated that low temperature of RF ablation at the target sites could facilitate rapid progression of residual hepatic VX2 carcinoma [12]. Cumulative evidence has demonstrated that residual tumor after RF ablation might exhibit an aggressive phenotype and unfavorable prognosis, and even change to sarcoma, which leads to the deterioration of patient's condition [12]. However, the mechanisms of rapid growth of residual HCC after RF ablation or the mediators which play a key role in the rapid growth are still poorly understood.

**Figure 1:**
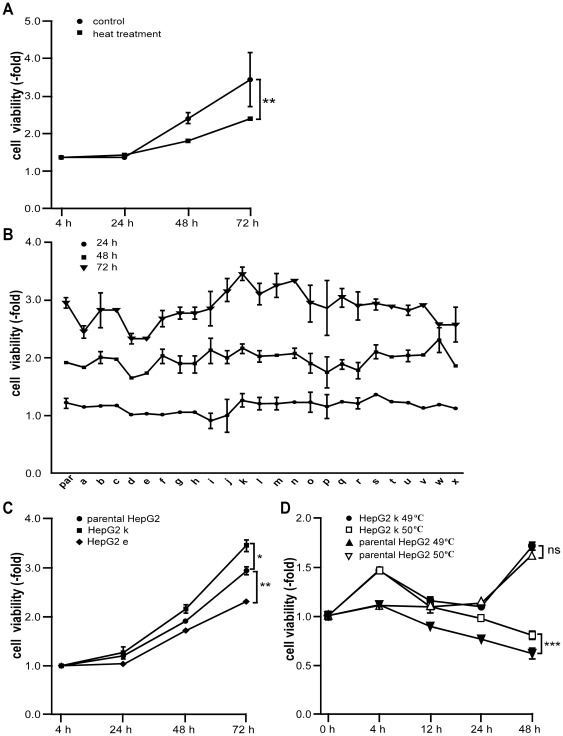
The viability of HepG2 cells and sublines derived from HepG2 cells after hyperthermia. (A) HepG2 cells were cultured after 47°C heat treatment. The 24 h, 48 h and 72 h cell viability of HepG2 cells with or without 47°C heat treatment were measured using MTT assay. (B) Twenty-four sublines were established after 47°C heat treatment for 10 min as described in the method. The 24 h, 48 h, and 72 h viability was evaluated by MTT assay after 24 sublines were established. par, parental HepG2 cells; a–x, sublines derived from the HepG2 cells. (C) The 24 h, 48 h and 72 h viability of representative sublines of HepG2 cells were evaluated by MTT assay. (D) Parental HepG2 and HepG2 k cells were treated with 49°C or 50°C 10 min. The 4 h, 12 h, 24 h and 48 h cell viability were measured by MTT assay. *, *P*<0.05; **, *P*<0.01; ***, *P* <0.001. Data are the representative results of three independent experiments. The coefficients of variation (CV) of all assays were shown in Supporting Information S1.

**Figure 2:**
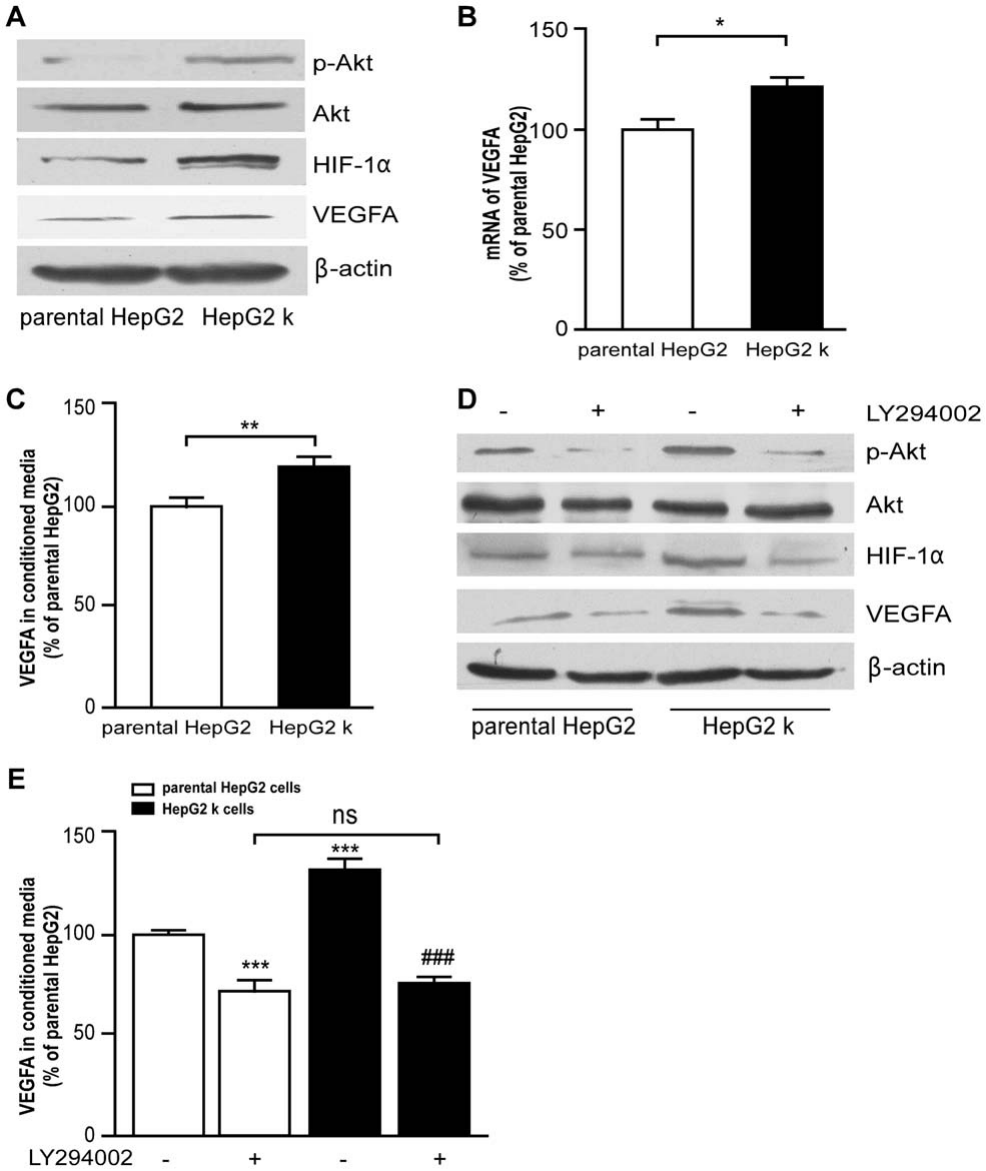
HepG2 k cells express elevated level of VEGFA. (A) Expression of p-Akt, HIF-1α and VEGFA in parental HepG2 and HepG2 k cells were detected by western blot analysis. (B) The mRNA expression of VEGFA in parental HepG2 and HepG2 k cells was assayed using real time PCR. *, *P*<0.05. (C) VEGFA concentration in conditioned media from parental HepG2 and HepG2 k cells was measured by ELISA analysis. **, *P*<0.01. (D) The expression of HIF-1α and VEGFA in parental HepG2 and HepG2 k cells treated with or without LY294002 (20 µM) for 24 h were analyzed by western blot analysis. (E) VEGFA concentration in conditioned media was detected by ELISA analysis. ***, *P*<0.001 versus parental HepG2 cells control respectively; ^###^, *P*<0.001 versus HepG2 k cells; ns, no significance.

**Figure 3:**
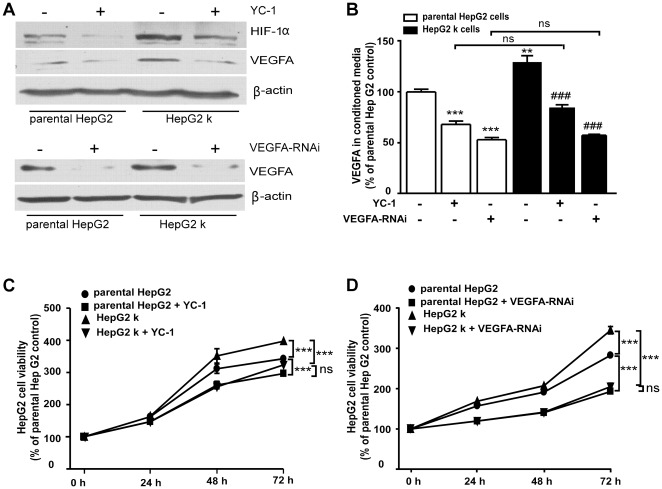
YC-1 and VEGFA siRNA inhibit HepG2 cell viability. The parental HepG2 and HepG2 k cells were treated with or without YC-1(5 µM) or transfected with or without VEGFA siRNA. (A) The expression of HIF-1α and VEGFA was detected by western blot analysis. (B) VEGFA concentration in conditioned media was detected by ELISA analysis. **, *P*<0.01, ***, *P*<0.001 versus parental HepG2 cells control respectively; ^###^, *P*<0.001 versus HepG2 k cells. (C) Parental HepG2 and HepG2 k cells were transfected with or without VEGFA siRNA for 24 h, 48 h, and 72 h, cell viability was measured by MTT assay. ***, *P*<0.01; ns, no significance. (D) Parental HepG2 and HepG2 k cells were treated with or without YC-1 (5 µM) for 24 h, 48 h, and 72 h, cell viability was measured by MTT assay. ***, *P*<0.001; ns, no significance.

**Figure 4:**
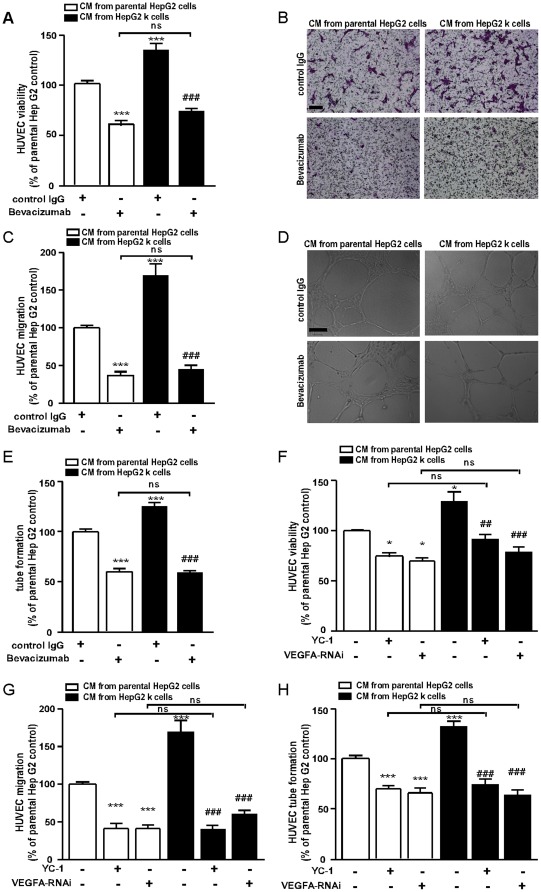
The enhanced pro-angiogenic ability of HepG2 k is in a HIF-1α/VEGFA dependent manner *in vitro*. (A) HUVECs were treated with conditioned media from parental HepG2 or HepG2 k cells with addition of bevacizumab (0.5 mg/ml) or control IgG for 24 h. Cell viability were quantified by MTT assay. (B–C) HUVEC migration *in vitro* in response to conditioned media from HepG2 k or parental HepG2 cells with addition of bevacizumab (0.5 mg/ml) or control IgG was assayed after 12 h. The number of migrated cells was quantified by counting 10 random fields at ×100 magnification. (D–E) HUVEC tube formation in response to conditioned media from HepG2 k or parental HepG2 cells with addition of bevacizumab (0.5 mg/ml) or control IgG after 20 h was assayed. The length of tube was evaluated by counting 10 random fields at ×100 magnification. (F–H) The parental HepG2 and HepG2 k cells were treated with or without VEGFA siRNA or 5 µM YC-1, and the conditioned media was collected. The effect of various conditioned media on HUVEC 24 h viability was quantified by MTT assay. The effect of various conditioned media on HUVEC migration for 12 h was assayed. The number of migrated cells was quantified by counting 10 random fields at ×100 magnification. The effect of various conditioned media on HUVEC tube formation for 20 h was assayed. The tube length was evaluated by counting 10 random fields at ×100 magnification. *, *P*<0.05, ***, *P*<0.001 versus parental HepG2 cell control; ^##^, *P*<0.01, ^###^, *P*<0.001 versus HepG2 k cells control; ns, no significance.

**Figure 5:**
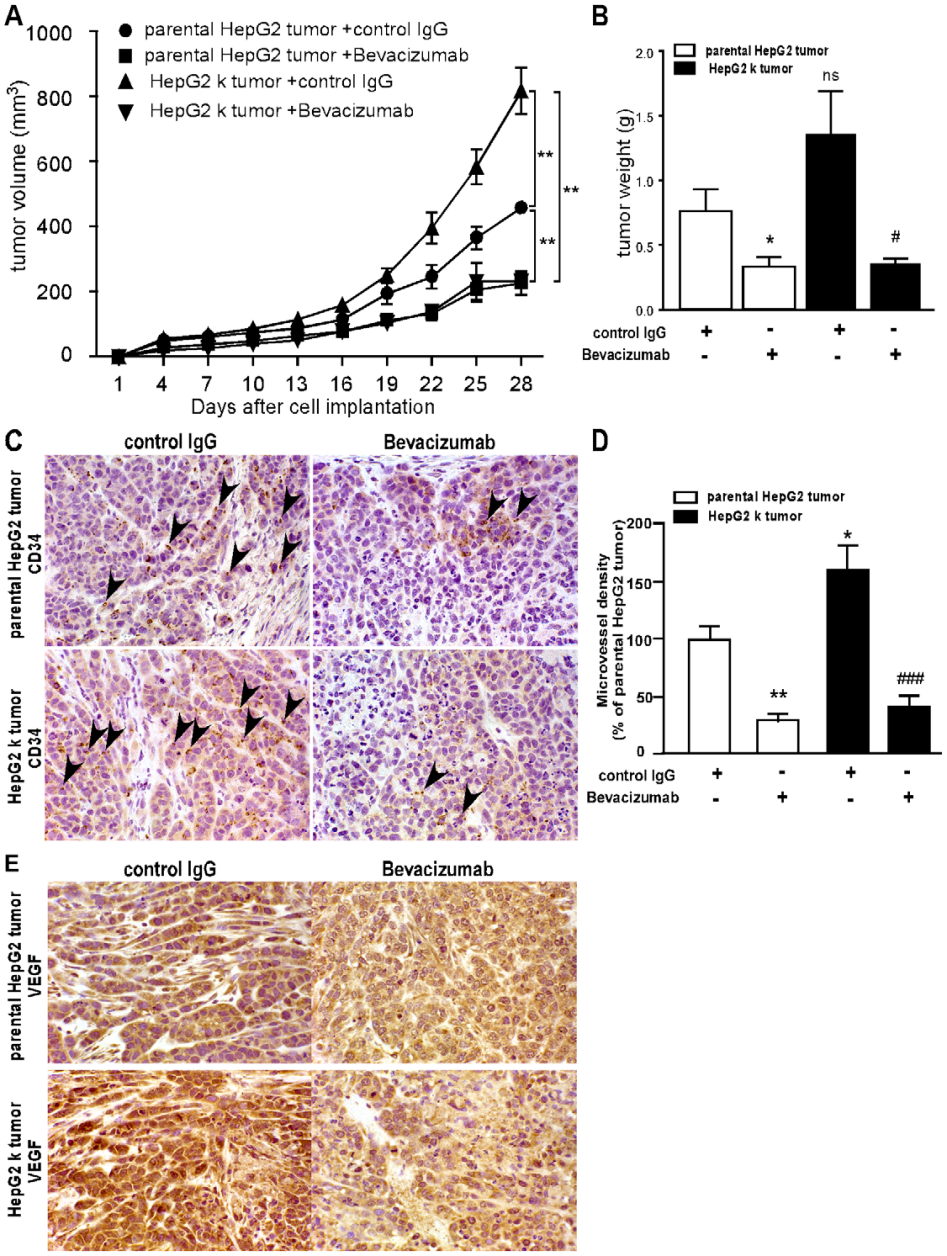
Bevacizumab impaired the tumor growth and angiogenesis in tumor-bearing mice *in vivo*. 2×10^6^ HepG2 k or parental HepG2 cells in cells in 200 µl phosphate buffered saline were injected by subcutaneous injection to obtain s. c. tumors. (A) Average tumor volume is shown for the HepG2 tumors. Parental HepG2 tumor with control IgG (n = 5), Parental HepG2 tumor with Bevacizumab (n = 5), HepG2 k tumor with control IgG (n = 5) and HepG2 k tumor with Bevacizumab (n = 5). **, *P*<0.01. (B) Mice were killed after 28 days implantation and the tumor tissues were removed and weighed. *, *P*<0.05. (C–D) Twenty-eight days after implantation, the numbers of new microvessels marked with CD34 (arrow heads) in the subcutaneous tumors were quantified by performing new vessel counts of 10 random fields at ×400 magnification. ^*^, *P*<0.05, ^**^, *P*<0.01 versus parental HepG2 tumor respectively; ^###^, *P*<0.001 versus HepG2 k tumor. (E) Immunohistochemistry analysis of the expression of VEGF in implanted tumors.

Hypoxia inducible factor-1 alpha (HIF-1α), a master regulator of essential adaptive responses to hypoxia, is highly expressed in hypoxic conditions and maintains a low concentration in normoxic condition [13]. HIF-1α is generally more pronounced in aggressive tumors [14] and can be an independent predictor of poor prognosis in HCC [15]. HIF-1α plays a major role in the development of a characteristic tumor phenotype influencing growth rate, angiogenesis, invasiveness, and metastasis [16]. Angiogenesis, which plays a critical role in tumor formation and maintenance [17], is the most significant because it is essential for the other biological characteristics [18]. A great number of angiogenesis associated genes are directly induced by HIF-1α, such as NOS (nitric oxide synthases), angiogenic and vascular growth factors (VEGF) and urokinasetype plasminogen activator receptor (uPAR). Vascular endothelial growth factor (VEGF) family, of structurally related molecules including VEGFA, VEGFB, VEGFC, VEGFD and placental growth factor (PLGF), is one of the most potent angiogenic factors expressed in various human cancers [19]. Studies have shown that VEGF is frequently expressed in HCC [20]. The expression of VEGF in HCC tissue or serum indicates the vascular invasion and metastasis of HCC, reduced median survival, and the recurrence of HCC after treatments [21]–[30]. Bevacizumab, a humanized monoclonal antibody directed against VEGFA ligand to prevent endothelial cell receptor activation, was the first anti-angiogenesis agent to attain approval from the United States Food and Drug Administration (FDA) [31]. Bevacizumab has been used not only in colorectal cancer, lung cancer, breast cancer, glioblastoma and renal cell carcinoma, but also in HCC [31]–[33]. However, whether bevacizumab can be used routinely to prevent the rapid growth of residual HCC after RF ablation is still not clear.

We hypothesized that insufficient RF ablation could promote proliferation and angiogenesis of residual HCC, which played a key role in the rapid growth of residual HCC after RF ablation. In this study, we sought to determine whether hyperthermia could directly generate a subline of HepG2 cells with the rapid proliferation and enhanced pro-angiogenic effect through a HIF-1α/VEGFA dependent mechanism.

## Materials and Methods

### Ethics Statement

The animal experiments were approved by the Animal Care Committee of Capital Medical University, Beijing, China and were performed in accordance with the institutional guideline.

### Materials

The PI3K/Akt inhibitor LY294002, the HIF-1α inhibitor YC-1, 3-(50-hydroxy methyl-20-furyl)-1-benzylindazole and MTT, 3-[4,5-dimethylthiazol-2-yl]-2,5-diphenyl tetrazolium bromide were purchased from Sigma-Aldrich (St. Louis, MO). Bevacizumab was purchased from Roche (Shanghai, China). Lipofectamine RNAiMIX reagent was obtained from Invitrogen (Carlsbad, CA). Antibodies used in the present study include the following: The rabbit polyclonal anti-CD34, anti-VEGFA and anti-β-actin antibodies were purchased from Boster (Wuhan, China). The rabbit monoclonal anti-phospho-Akt antibody was provided by Cell Signaling Technology (Danvers, MA). The anti-Akt antibody was purchased from Santa Cruz Biotechnology (Santa Cruz, CA). HRP-goat-anti-rabbit IgG and HRP-goat-anti-mouse IgG were purchased from MBL (Nagoya, Japan).

### Cell lines and cell culture

HepG2 cells, a human hepatoma cell line, was brought from Cell Resource Center, Chinese Academy of Medical Sciences, Peking Union Medical College, and cultured in Dulbecco's modified Eagle's with high glucose supplement (DMEM, GIBCO, UK) containing 10% fetal bovine serum (FBS) in a humidified incubator at 37°C with an atmosphere of 5% CO_2_. Human umbilical vein endothelial cells (HUVECs) were isolated by collagenase digestion of umbilical veins from fresh cords. The cells were plated on gelatin-coated culture dishes in Endothelial Cell Medium(ECM; Science, USA) consisting of 500 ml of basal medium, 25 ml of fetal bovine serum, 5 ml of endothelial cell growth supplement and 5 ml of penicillin and streptomycin solution and then cultured in a humidified incubator at 37°C with an atmosphere of 5% CO_2_. HUVECs were used at passages 2–5.

### Heat treatment of HepG2 cells and establishment of sublines

The sublines of HepG2 cells after heat treatment were established as described previously [34]. HepG2 cells were seeded into the 96-well plate. And after 24 h of incubation, in some experiments, the plates were sealed and submerged in a water bath set to 47°C for 10 min. Thereafter, the plates were maintained in the incubator at 37°C with an atmosphere of 5% CO_2_. The 24 h, 48 h and 72 h cell viability with or without heat treatment was measured by MTT assay. To establish the sublines, cells were cultured in the 24-well plate for the above heat treatment. Then cells were cultured until subconfluent and passaged sequentially into the 6-well plate and the 10 cm dish. After two passages in the 10 cm dish, these cells were used as 24 sublines.

### MTT assay

The cell viability was evaluated by MTT assays. Cells were seeded at a concentration of 2×10^3^/well in 96-well plates. MTT solution was added to each well at final concentration of 0.5 mg/ml and incubated for 4 h. At the end of incubation, formazan crystals resulting from MTT reduction were dissolved by addition of 150 µl dimethyl sulfoxide (DMSO) per well. The optical density was read at 570 nm with a plate reader (model 550, BioRad, USA). The average values were determined from different wells of the representative assay.

### Heat sensitivities of parental HepG2 and HepG2 k cells

Parental HepG2 and HepG2 k cells were seeded at a concentration of 5×10^3^/well in 96-well plates. After 12 h of incubation, parental HepG2 and HepG2 k cells were treated by 49°C or 50°C for 10 min. The 4 h, 12 h, 24 h and 48 h cell viability were measured by MTT assay as described above.

### Western blot

Cells were lysed using cell lysis buffer (150 mM NaCl, 50 mM Tris-HCl, pH 8.0, 0.1% SDS, 1% Triton X-100) containing protease inhibitors. Equivalent amounts of whole cell extracts were subjected to SDS-PAGE gel and transferred to nitrocellulose membranes. The membranes were blocked with 5% non-fat milk for 2 h and then incubated with respective primary antibody for 5 h followed by the incubation of the appropriate HRP-conjugated secondary antibody for 2 h at room temperature. Blots were visualized with an ECL detection kit (Pierce, USA) and analyzed using Quantity One 1-D Analysis Software (Bio-Rad, CA).

### Real-time PCR assay for VEGFA

Real-time PCR was performed to determine mRNA level of VEGFA. Total mRNA was extracted using the TRIzol reagent (Invitrogen, USA) and reverse transcription was performed using an RT-PCR kit (TransGen Biotech, China). Real-time experiments were conducted on a DNA Engine Opticon System (MJ research Inc, USA) using SYBR Green PCR Master Mix kit in triplicate specific primers. The sequences of primers to determine the expression of the target gene were listed as follows: VEGFA [5′-TTCAAGCCATCCTGTGC-3′ (forward); 5′-TGCTCTATCTTTCTTTGGTCTGC-3′ (reverse)] and Glyceraldehydes 3-phosphate dehydrogenase (GAPDH) [5′-CGGAGTCAACGGATTTGGTCGTAT-3′ (forward); 5′-AGCCTTCTCCATGGTGGTGAAGAC-3′ (reverse)]. The PCRs consisted of 5 min at 95°C followed by 40 cycles of denaturation for 30 s at 95°C, annealing for 30 s at 56°C and a primer extension for 30 s at 72°C. The comparative CT method was used to quantitate the expression of VEGFA using GAPDH as the normalized control.

### siRNA Knockdown of VEGFA

The VEGFA siRNA and scramble siRNA [scramble siRNA sequence: sense strand (5′-UUCUCCGAACGUGUCACGUTT-3′) and antisense strand: (5′-ACGUGACACGUUCGGAGAATT-3′); VEGFA siRNA sequence: sense strand (5′-CCGAAACCAUGAACUUUCUTT-3′) and antisense strand: (5′-AGAAAGUUCAUGGUUUCGGTT-3′)] were synthesized by Shanghai GenePharma Co (Shanghai, China). HepG2 cells were plated into 6-well plates and allowed to grow to sub-confluent. Cells were transiently transfected with the siRNA with lipofectamine RNAiMIX reagent (Invitrogen, Carlsbad, CA) in OPTI-MEM medium (Gibco) for 12 h, and then incubated and used for further experiments.

### Collection of the conditioned medium

HepG2 cells were transiently transfected with the VEGFA siRNA or scramble siRNA, or treated with YC-1 (5 µM) or vehicle for 12 h, and then incubated in DMEM with 0.1% BSA for 14 h followed by collection of the conditioned medium. The medium was spun down at 3000 rpm, 20 min, and the supernatant was collected and stored at −80°C. In the experiments of bevacizumab blocking assay, bevacizumab and control IgG (final, 0.5 mg/ml) were added into conditioned media 30 min before further experiment.

### Quantification of VEGFA in the conditioned media

VEGFA concentrations in the conditioned media were quantified using an enzyme-linked immunosorbent assay (ELISA) kit (Dakewe Biotech, China) according to the manufacture's instructions. We collected the total cell protein to assess the different cell numbers of the different group. Equal volume of lysis buffer was added before we extracted the total cellular protein, then we performed bicinchoninic acid (BCA) assay to evaluate the protein concentration. Thereafter, the VEGFA concentration was normalized to the total cellular protein.

### Cytotoxicity of bevacizumab on HUVECs

HUVECs (1×10^4^/well) were seeded into gelatin-coated 96-well plates and allowed initially to attach for 24 h. Bevacizumab was added to the wells at final concentration of 0.5 mg/ml. 24 h cell viability was performed by MTT assay as described above.

### HUVEC viability assays

HUVECs were seeded into gelatin-coated 96-well plates. After 24 h incubation, the ECM was removed and various conditioned media were added to the wells. HUVEC viability was evaluated by MTT assay as described above. The relevant effect of conditioned media was normalized to the total cellular protein.

### HUVEC migration assay

Quantitative cell migration assays were performed using a modified Boyden chamber (Minicell, Millipore, USA) with 8.0-µm pore polycarbonate filter inserts in 24-well plates as described before [35]. Briefly, the lower chamber was filled with various conditioned media. HUVECs (5×10^4^ cells/well) in serum-free medium were added into the upper chamber. The cells were allowed to migrate for 12 h at 37°C. The non-migrated cells were removed from the upper surface of the membrane by scraping with a cotton swab, and the migrated cells were fixed with methanol, stained with crystal violet, and photographed under an inverted microscope (Nikon, Japan). Migration was assessed by counting the number of stained cells from 10 random fields at ×100 magnification. The relevant effect of conditioned media was normalized to the total cellular protein.

### HUVEC tube formation assays

HUVECs (1×10^4^/well) resuspended by various conditioned media were added to Matrigel coated 96-well plates and incubated at 37°C for 20 h. HUVECs were photographed under an inverted microscope (Nikon, Japan). Tube formation was assessed by counting the length of tube from 10 random fields at ×100 magnification. The relevant effect of conditioned media was normalized to the total cellular protein.

### Xenograft assays

Male BALB/c nude mice (5 weeks old) were purchased from Vital River Laboratories and maintained in the Laboratory Animal Center of Capital Medical University. 20 mice were randomized into 4 groups and housed in laminal-flow cabinets under specific pathogen-free (SPF) conditions. 2×10^6^ HepG2 k or parental HepG2 cells in 200 µl phosphate buffered saline were implanted by subcutaneous injection to obtain s. c. tumors. After establishment, tumor-bearing mice were treated with bevacizumab or control IgG (5 mg/kg i.p. qd) every day. The tumor growth was monitored by periodic caliper every 3 days, and the tumor volume was calculated according to the formula tumor volume = (largest diameter × perpendicular^2^)/2. After 4 weeks, mice were euthanized and tumor tissues were removed for fixation in the 4% paraformaldehyde solution.

### Immunohistochemical analysis

Tumor tissues from mouse model (n = 20) were embedded into paraffin. Paraffin-embedded tissue sections were cut into standard 6-µm sections, deparaffinaged in xylene and rehydrated through graded alcohol solutions. Antigen retrieval was performed 10 min at 93°C in EDTA (10 mmol/l, PH 8.0) in a water bath. Endogenous peroxidases were inactivated by immersing the sections in 0.3% hydrogen peroxide for 12 min. The sections were incubated at 37°C with primary antibodies contained mouse monoclonal mouse monoclonal anti-VEGF (dilution 1∶50) or rabbit polyclonal anti-CD34 (dilution 1∶100) for 2 h in a humidified chamber, then incubated with the appropriate HRP-conjugated secondary antibody for 40 min at 37°C. Staining results were viewed under light microscope (Olympus, Leeds Precision Instruments) and pictures were taken with an imaging program.

### Statistical analysis

As indicated, Student's t test or ANOVA test was used for comparison of two groups or four groups using GraphPad Prism (GraphPad Software Inc., La Jolla, CA). P<0.05 was set as the level of statistical significance.

## Results

### Heat treatment can generate sublines from HepG2 cells with higher viability

To monitor the potential effect of hyperthermia on the tumor growth, initially we studied the cell viability at 24 h, 48 h, and 72 h of HepG2 cells after the treatment of 47°C for 10 min. It was found that the cell viability of HepG2 cells after 47°C heat treatment significantly decreased compared with no heat treatment due to cell apoptosis and death (*P*<0.001; Figure 1A). To examine the cell viability of survived HepG2 cells after heat treatment, HepG2 cells were cultured in the 24-well plate for 47°C heat treatment and survived cells were continuously cultured until 24 sublines were generated as described in the method. After 24 sublines were generated, the cell viability of sublines was tested at 24, 48, and 72 hour. It was found that the sublines exhibited significant difference in cell viability compared with parental HepG2 cells, especially HepG2 k, showing nearly 18.8±3.1% higher (*P*<0.001; Figure 1B and C). Furthermore, we determined the difference between parental HepG2 cells and HepG2 k cells in heat resistance. HepG2 k cells showed significant heat tolerance compared with parental HepG2 cells after 50°C 10 min treatment (Figure 1D). Therefore, it appeared that heat treatment could kill HepG2 cells partially and generate a subline derived from parental HepG2 cells with higher viability and heat resistance. Then we selected HepG2 k cells for further studies.

### VEGFA expression is up-regulated in HepG2 k cells involving PI3K/Akt/HIF-1α/VEGFA signaling pathway

We sought to determine the molecular discrepancy between parental HepG2 cells and HepG2 k cells. Western blot demonstrated that Akt phosphorylation and protein levels of HIF-1α and VEGFA were increased in HepG2 k cells compared with parental HepG2 cells (Figure 2A). The mRNA level of VEGFA in HepG2 k cells increased 24±5.4% compared with parental HepG2 cells (*P*<0.05; Figure 2B). The ELISA results showed that 18.2±2.6% more VEGFA was secreted from HepG2 k cells than that from parental HepG2 cells (*P*<0.01; Figure 2C). Subsequently, we observed that both specific PI3K/Akt inhibitor LY294002 and HIF-1α inhibitor YC-1 were able to decrease HIF-1α, VEGFA expression, and secreted VEGFA in parental HepG2 cells and HepG2 k cells (Figure 2D,E and 3A,B). Our findings demonstrated that increased VEGFA expression in HepG2 k cells was regulated involving the PI3K/Akt/HIF-1α/VEGFA pathway.

### YC-1 and VEGFA siRNA inhibit HepG2 cell viability

To investigate the role of HIF-1α and VEGFA in the difference of cell viability between parental HepG2 cells and HepG2 k cells, YC-1 and VEGFA siRNA were used. The viability of parental HepG2 cells and HepG2 k cells at 24, 48, and 72 h after YC-1 treatment and VEGFA siRNA transfection was significantly lower than those of the control group (Figure 3C and D). Meanwhile, YC-1 treatment or VEGFA siRNA transfection eliminated the difference of viability between parental HepG2 cells and HepG2 k cells (Figure 3C and D).

### HepG2 k cells promotes angiogenesis through HIF-1α/VEGFA

We further explored the difference of HepG2 k cells and parental HepG2 cells in promoting the angiogenesis and the roles of HIF-1α and VEGFA in that process. It was found that the effect of the conditioned media (plus control IgG) from HepG2 k cells on HUVEC viability (Figure 4A), migration (Figure 4B,C), and tube formation (Figure 4D,E) increased by 36.7±9.6%, 44.3±16.5% and 33.3±11.9% respectively, compared with that from parental HepG2 cells (all at *P*<0.001). The addition of bevacizumab showed no cytotoxicity on HUVECs (Figure S1 in Supporting Information S1) and blocked the pro-angiogenic effect of the conditioned media on HUVEC viability, migration and tube formation (all at *P*<0.001; Figure 4A–E). In addition, bevacizumab eliminated the significant difference in pro-angiogenic effect of the conditioned media between HepG2 k cells and parental HepG2 cells (Figure 4A–E). To further determine the effect of HIF-1α/VEGFA mechanism in angiogenesis, HIF-1α inhibitor YC-1 and VEGFA siRNA were used to decrease the expression and secretion of VEGFA (Figure 3A and B). It was found that HUVEC viability, migration and tube formation were significantly inhibited by the conditioned media from HepG2 cells with YC-1 treatment or knockdown of VEGFA (Figure 4F,G and H). Moreover, YC-1 and VEGFA siRNA eliminated the significant difference in pro-angiogenic effect of the conditioned media between HepG2 k cells and parental HepG2 cells (Figure 4F,G and H).

### HepG2 k subline cells have higher tumor growth and angiogenesis *in vivo*, and Bevacizumab can inhibit them

To confirm the results *in vitro*, we examined the growth of the HepG2 k tumor and parental HepG2 tumor, and the effects of bevacizumab *in vivo*. After 28 days of treatment, the group of HepG2 k tumor with control IgG showed increased tumor volume in comparison to the group of parental HepG2 tumor with control IgG *(P*<0.01); groups with treatment of bevacizumab showed decreased tumor volume in comparison to groups with treatment of control IgG (*P*<0.01); the treatment of bevacizumab significantly diminished the difference of the tumor growth between HepG2 k tumor and parental HepG2 tumor (Figure 5A). Similarly, the weights of the HepG2 k tumor xenografts are higher than those of the parental HepG2 tumor, but with no statistical significance. Additionally, the weights of groups injected with bevacizumab were markedly decreased in comparison to that of the groups with control IgG (*P*<0.05; Figure 5B). Quantification of CD34-marked microvessel density (MVD) in tumor xenograft samples indicated that samples derived from the group of HepG2 k tumor with control IgG revealed a 59.4±40.0% higher angiogenesis in comparison to the group of parental HepG2 tumor with control IgG (*P*<0.05; Figure 5C and D). And bevacizumab inhibited the angiogenesis of parental HepG2 tumor and HepG2 k tumor significantly (**, *P*<0.01; ###, *P*<0.001; Figure 5C and D). Immunohistochemistry analysis showed that, in the groups treated with control IgG, the expression of VEGF in HepG2 k tumor was higher compared with that in parental HepG2 tumor. And bevacizumab significantly inhibited the expression of VEGF in HepG2 k tumor and parental HepG2 tumor (Figure 5E).

## Discussion

The phenomenon of rapid growth of residual liver tumor after RF ablation has been observed in many clinical centers. Our previous study demonstrated that residual tumor was prone to proliferation, invasion and metastasis when the local ablative temperature was not sufficiently high [12]. However, whether the angiogenesis which may be induced by hyperthermia produced by RF ablation on HCC cells plays a role in the rapid growth of residual HCC after RF ablation has remained undetermined.

*In vitro* we simulated the growth pattern of the hepatoma cells which were not killed after treatment of hyperthermia generated by RF ablation. In the study of Obara et al, one of HepG2 sublines showed more than 100% increase in proliferation, marked heat tolerance and no obvious difference in invasive capacities compared with parental HepG2 cells [34]. But the mechanism of HepG2 with rapid proliferation was not further investigated. In this study, we found that cell viability of HepG2 cells was inhibited after the transient heat exposure of 47°C, which was consistent with the previous study [36], and we also established the sublines of HepG2 cell which survived the 47°C heat treatment for 10 min. Among the sublines, HepG2 k cells with the highest viability showed 18.8±3.1% increase in viability compared with parental HepG2 cells. Although the increased percentage of the subline cell viability in comparison to the parental HepG2 cells was not corresponding to the previous study, we both revealed that the hyperthermia can generate a subline with high viability and heat tolerance. Moreover, cumulative evidence has shown that suppression of VEGF and HIF-1α inhibited carcinoma cell proliferation [37]–[40]. Our further findings demonstrated that the higher viability of the subline was mediated involving HIF-1α/VEGFA pathway.

According to Forkman's reports, the generation of a tumor mass requires tumor cell proliferation plus angiogenesis. Tumor cell proliferation alone, in the absence of angiogenesis, can give rise to dormant, microscopic tumors of ∼1 mm^3^ or less, but these in situ cancers are harmless to the host [41]. HCC is also a highly vascular tumor, and the angiogenesis plays a considerable role in its development and progression [42]. Similarly, our study revealed that both the pro-angiogenic effect of HepG2 k cells *in vitro* and the angiogenesis of HepG2 k tumor *in vivo* were stronger than that of parental HepG2 and that of parental HepG2 tumor respectively.

VEGF, whose most important member is VEGFA, is the most potent angiogenic factor and plays a key role in tumor associated angiogenesis and hyper-permeability [19], [43], [44]. Our previous study demonstrated that insufficient RF ablation due to low temperature at the target sites significantly increased the expression of VEGF of residual VX2 tumor [12]. HIF-1 is a transcription activator of the VEGF promoter. HIF-1α, a component of HIF-1, can be induced by hypoxia or mutations of PTEN, VHL, succinate dehydrogenase (SDH), or fumarate hydratase (FH), as well as by the activation of ERBB2, SRC, endothelin-1 (ET-1), the RAS/MARK pathway, and the phosphoinositide 3-kinase (PI3K)-Akt-mTOR pathway. Moreover, HIF-1α can be stabilized by reactive oxygen species (ROS) through blocking the PHD activities [45], [46]. Based on these previous researches, we further explored the underlying molecular mechanism of the enhanced pro-angiogenic effect of HepG2 k cells *in vitro* and the angiogenesis of HepG2 k tumor *in vivo*. We found that the enhanced Akt phosphorylation and high protein levels of HIF-1α and VEGFA caused the subline to show high viability, which was not reported previously. Additionally, after we performed HIF-1α inhibitor YC-1 treatment and VEGFA siRNA transfection to decrease the VEGFA concentration in the conditioned media, it was found that the pro-angiogenic effect of the conditioned media was impaired correspondingly and the difference in pro-angiogenic effect of HepG2 k and parental HepG2 also diminished. Moreover, bevacizumab to block the effect of VEGFA in the conditioned media also showed the similar results. Considering the findings above, we postulated that the hyperthermia-induced subline exerted its stronger pro-angiogenic effect through enhanced PI3K/Akt/HIF-1α/VEGFA signaling pathway. These novel findings could be a potential mechanism associated with the rapid growth of the residual tumor after radiofrequency (RF) ablation and therefore could bear important therapeutic implications.

Anti-VEGF therapies are important in the treatment of certain cancers. Especially, there have been many studies indicative of the potential therapeutic benefit of bevacizumab in combination anticancer therapy [47]–[51]. However, whether bevacizumab can be used to control the rapid progression of residual HCC after RF ablation has not been reported yet. Our study demonstrated that bevacizumab treatment reduced the pro-angiogenic effect of conditioned media from HepG2 k cells. Additionally, bevacizumab significantly inhibited the tumor growth and angiogenesis of HepG2 k tumor *in vivo*, and bevacizumab eliminated the difference of tumor growth and angiogenesis between HepG2 k tumor and parental HepG2 tumor. Therefore, we may provide the support information of using bevacizumab to prevent the rapid progression of residual HCC after RF ablation.

Our findings were that hyperthermia induced hepatoma cells to generate a subline with enhanced PI3K/Akt/HIF-1α/VEGFA signaling pathway, high viability and dys-regulated pro-angiogenic effect, which explored the underlying mechanism and potential therapeutic target of the rapid growth of residual HCC after RF ablation. Obviously, further researches are needed in the future to clarify the disease of rapid growth of residual HCC after RF ablation.

## Supporting Information

Supporting Information S1 **Contains Figure S1, The effect of bevacizumab on HUVEC cytotoxicity, and Table S1, The coefficients of variation (CV) of all assays.**
(DOC)
