# Supplementary material for: Insufficient Radiofrequency Ablation Promotes Angiogenesis of Residual Hepatocellular Carcinoma via HIF-1α/VEGFA
Source: PLoS One. 2012 May 15;7(5):e37266. doi: 10.1371/journal.pone.0037266 (PMC3352883; doi:10.1371/journal.pone.0037266)
Supplement: Supporting Information S1 — Contains Figure S1, The effect of bevacizumab on HUVEC cytotoxicity, and Table S1, The coefficients of variation (CV) of all assays. (DOC) [file pone.0037266.s001.doc]

**Supporting Information S1**

**Figure S1 The effect of bevacizumab on HUVEC cytotoxicity.** HUVECs were treated with or without 0.5 mg/ml bevacizumab for 24 h, and HUVEC vitality was assessed using MTT assay. ns, no significance.

**
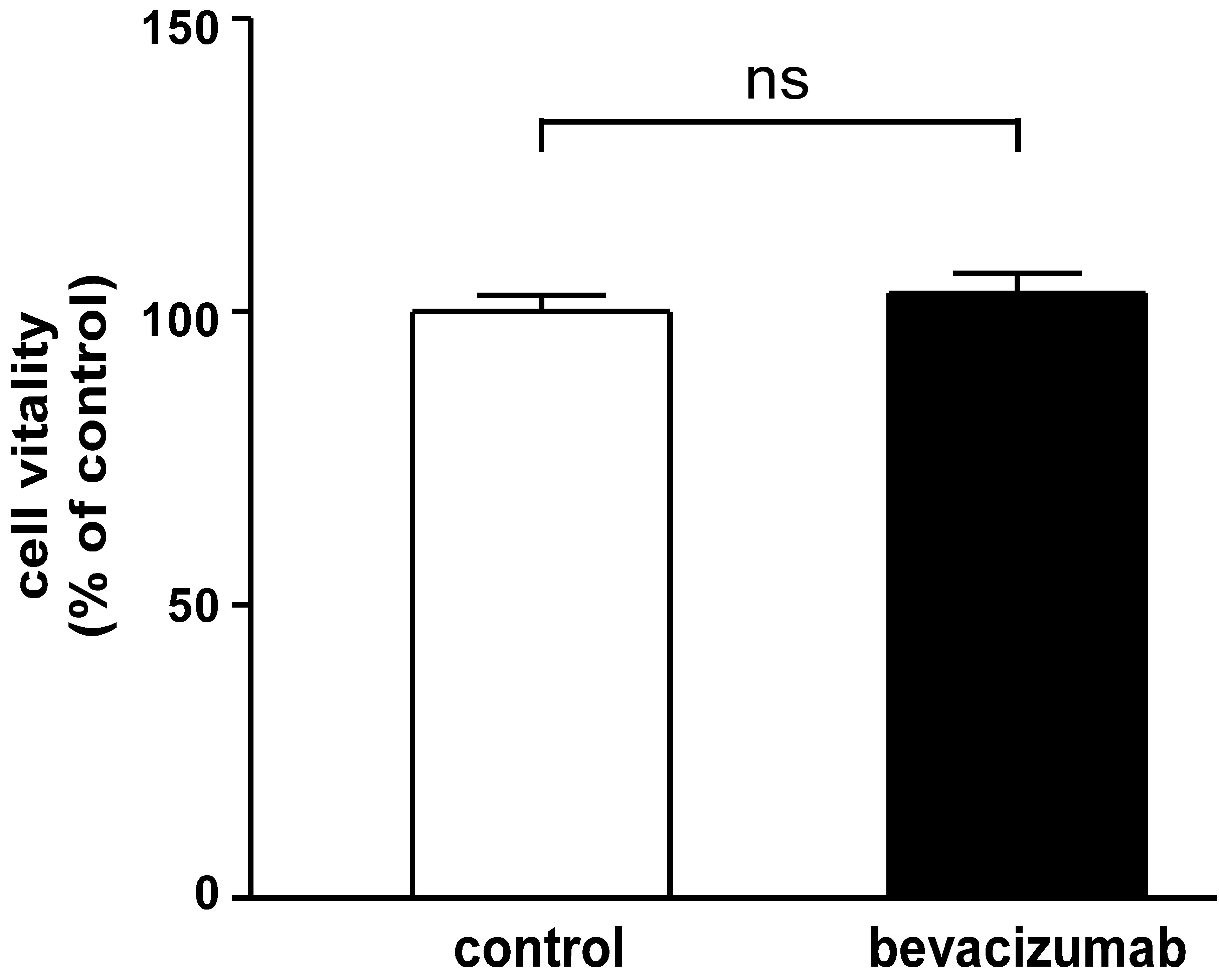
**

**Table S1 The coefficients of variation (CV) of all assays.**

CV in Fig 1 A

|  | 37℃ | 47℃ |
| --- | --- | --- |
| CV | 7.8% | 20% |

CV in Fig 1B

|  | par | a | b | c | d | e | f | g | h | i | j | k |
| --- | --- | --- | --- | --- | --- | --- | --- | --- | --- | --- | --- | --- |
| CV | 5.3% | 4.15% | 10.6% | 1.6% | 3.7% | 2.5% | 5.1% | 3.7% | 4.7% | 10.7% | 6.2% | 3.3% |

| l | m | n | o | p | q | r | s | t | u | v | w | x |
| --- | --- | --- | --- | --- | --- | --- | --- | --- | --- | --- | --- | --- |
| 6.1% | 6.3% | 1.6% | 10.8% | 16.5% | 5.4% | 8.5% | 3% | 2.3% | 3% | 1.8% | 1.9% | 11.8% |

.

CV in Fig 1 D

|  | par HepG2 49℃ | par HepG2 50℃ | HepG2 k 49℃ | HepG2 k 50 ℃ |
| --- | --- | --- | --- | --- |
| CV | 5.5% | 1.8% | 4.8% | 1.1% |

CV in Fig 2 B

|  | par HepG2 | HepG2 k |
| --- | --- | --- |
| CV | 10.8% | 4.4% |

CV in Fig 2 C

|  | par HepG2 | HepG2 k |
| --- | --- | --- |
| CV | 3.7% | 1.6% |

CV in Fig 2 E

|  | par HepG2 | ParHepG2  +LY294002 | HepG2 k | HepG2 k+LY294002 |
| --- | --- | --- | --- | --- |
| CV | 1.3% | 14% | 4.9% | 2.9% |

CV in Fig 3 B

|  | par HepG2 control | par HepG2 +VEGFA siRNA | par HepG2  +YC-1 | HepG2 k  control | HepG2 k VEGF siRNA | HepG2 k +YC-1 |
| --- | --- | --- | --- | --- | --- | --- |
| CV | 4.5% | 6.7% | 8.5% | 9.0% | 2.5% | 7% |

CV in Fig 3 C

|  | par HepG2 | ParHepG2  +YC-1 | HepG2 k | HepG2 k+YC-1 |
| --- | --- | --- | --- | --- |
| CV | 3.3% | 1.2% | 1.1% | 2.3% |

CV in Fig 3 D

|  | par HepG2 | parHepG2  VEGFA siRNA | HepG2 k | HepG2 k  VEGFA siRNA |
| --- | --- | --- | --- | --- |
| CV | 1.7% | 3.9% | 5.3% | 8.5% |

CV in Fig 4 A

|  | par HepG2  control IgG | parHepG2  bevacizumab | HepG2 k  control IgG | HepG2 k  bevacizumab |
| --- | --- | --- | --- | --- |
| CV | 13.4% | 5.4% | 7% | 9.5% |

CV in Fig 4 C

|  | par HepG2  control IgG | parHepG2  bevacizumab | HepG2 k  control IgG | HepG2 k  bevacizumab |
| --- | --- | --- | --- | --- |
| CV | 7.9% | 19.9% | 22.1% | 26.7% |

CV in Fig 4 E

|  | par HepG2  control IgG | parHepG2  bevacizumab | HepG2 k  control IgG | HepG2 k  bevacizumab |
| --- | --- | --- | --- | --- |
| CV | 10.4% | 17% | 10.7% | 18.7% |

CV in Fig 4 F

|  | par HepG2 control | par HepG2 +VEGFA siRNA | par HepG2  +YC-1 | HepG2 k  control | HepG2 k VEGF siRNA | HepG2 k +YC-1 |
| --- | --- | --- | --- | --- | --- | --- |
| CV | 1.9% | 7.2% | 6.3% | 12.1% | 12.7% | 9.2% |

CV in Fig 4 G

|  | par HepG2 control | par HepG2 +VEGFA siRNA | par HepG2  +YC-1 | HepG2 k  control | HepG2 k VEGF siRNA | HepG2 k +YC-1 |
| --- | --- | --- | --- | --- | --- | --- |
| CV | 7.7% | 26.5% | 4% | 25.5% | 21.8% | 3.5% |

CV in Fig 4 H

|  | par HepG2 control | par HepG2 +VEGFA siRNA | par HepG2  +YC-1 | HepG2 k  control | HepG2 k VEGF siRNA | HepG2 k +YC-1 |
| --- | --- | --- | --- | --- | --- | --- |
| CV | 8.9% | 16.4% | 8.8% | 8.9% | 13.8% | 14.5% |

CV in Fig 5C

|  | par HepG2  control IgG | parHepG2  bevacizumab | HepG2 k  control IgG | HepG2 k  bevacizumab |
| --- | --- | --- | --- | --- |
| CV | 21.7% | 45.5% | 25.1% | 35.1% |

CV in Fig S1

|  | control | bevacizumab |
| --- | --- | --- |
| CV | 6.4% | 7.2% |
